# Comparison of *N*-butylcyanoacrylate glue and sutures for closure of inguinal skin incision after hernioplasty

**DOI:** 10.5339/qmj.2026.12

**Published:** 2026-03-17

**Authors:** Nandkishor D Shinde, Afia Kausar, Anup S Desai

**Affiliations:** Department of Surgery, Maharashtra Institute of Medical Sciences and Research (MIMSR), Latur, Maharashtra, India; Faculty of Medical Sciences, Department of Surgery, Khaja Bandanawaz University, Kalaburagi, Karnataka, India *Email: drnandkishorshinde@gmail.com

**Keywords:** Suture, *N*-butylcyanoacrylate, inguinal hernia, patient satisfaction, surgeon satisfaction, seroma

## Abstract

**Background:**

Skin closure techniques in inguinal hernia surgery significantly influence postoperative outcomes, including pain, healing, scar formation, and patient satisfaction. The aim of this study is to evaluate the efficacy of *N*-butyl-2-cyanoacrylate tissue adhesive as an alternative to conventional sutures for skin closure in inguinal hernia surgeries.

**Methods:**

A prospective cross-sectional study was conducted at a tertiary care center from November 2023 to October 2025. Seventy patients aged 18–60 years undergoing inguinal hernia repair were randomly assigned to two groups: Group 1 (sutures) and Group 2 (tissue adhesive). Exclusion criteria included recurrent or strangulated hernia, allergies to cyanoacrylate/formaldehyde, and immunocompromised states. Outcomes assessed included pain (VAS), wound healing (Hollander score), scar quality (Vancouver Scar Scale and Visual Analogue Scar Scale), complications, and satisfaction scores.

**Results:**

Group 2 showed significantly lower pain scores at early postoperative intervals (*p* < 0.05), shorter wound closure time (8.8 ± 1.4 min vs. 11.7 ± 2.6 min; *p* = 0.000), and reduced hospital stay (1.9 ± 0.8 days vs. 3.5 ± 1.6 days; *p* = 0.000). No infections were reported in either group. Chronic pain at 30 days was less frequent in Group 2 (*p* = 0.046). Wound healing and scar scores favored Group 2 on days 7 and 30 (*p* < 0.05). Patient satisfaction was higher in Group 2 across all follow-up visits, while surgeons preferred sutures (*p* < 0.05).

**Conclusion:**

*N*-Butyl-2-cyanoacrylate tissue adhesive offers superior early postoperative outcomes and higher patient satisfaction compared to sutures, although surgeons reported greater satisfaction with traditional methods. Tissue adhesive is a viable alternative for skin closure in inguinal hernia surgeries.

## 1. INTRODUCTION

The global burden of inguinal hernia has been estimated to be 7.7%, with the highest prevalence of 12.7% in Asia and the lowest in the Americas.^1^ Inguinal hernia repair surgeries are the most common elective surgeries performed. It is estimated that inguinal hernias affect approximately 15–20% of the general population.^2^

While the surgical technique itself has evolved significantly over the years, the method of skin closure remains a critical component of postoperative care.^3^ Traditionally, sutures have been the gold standard for closing surgical incisions due to their reliability, tensile strength, and cost-effectiveness. However, the emergence of tissue adhesives, such as *N*-butylcyanoacrylate glue (tissue glue), has introduced a promising alternative that may offer advantages in terms of patient comfort, cosmetic outcomes, and procedural efficiency. Sutures, however, have several disadvantages, including the risk of needle-stick injuries, the formation of suture marks, and the need for a follow-up visit to the hospital for suture removal. These disadvantages have led to the search for alternative methods of wound closure.^4^

*N*-Butylcyanoacrylate is a synthetic monomer that polymerizes rapidly upon contact with moisture, forming a strong bond that seals the wound edges. Its bacteriostatic properties, ease of application, and ability to eliminate the need for suture removal have made it an attractive option for skin closure across various surgical disciplines. In the context of inguinal hernioplasty, where incisions are typically superficial and linear, the use of tissue adhesive may reduce operative time, minimize postoperative pain, and enhance aesthetic results. It has been used for the closure of abdominal wounds, repair of traumatic lacerations, and mesh fixation in hernia repair.^4^^,^^5^

There are very few studies comparing the two methods—tissue adhesive and suture—for inguinal skin incision closure. This study aims to compare the efficacy, safety, and patient-centered outcomes of *N*-butylcyanoacrylate glue with those of conventional sutures for closing inguinal skin incisions following hernioplasty.

## 2. METHODS

The present prospective cross-sectional study was conducted at a tertiary care center from November 2023 to October 2025. All patients undergoing inguinal surgeries who were willing to participate, aged 18–60 years, irrespective of sex, were included in the study. Patients above 60 years often present with comorbidities, delayed wound healing, and altered pain perception, which could confound outcome measures. Therefore, the age limit was set to ensure homogeneity of the study population. Patients with strangulated and recurrent inguinal hernia, known allergies to cyanoacrylate, chronic infection, immunocompromised status, or connective tissue disorders were excluded from the study. The study was conducted only after obtaining approval from the ethical committee (No: IEC/2023/178). Before inclusion, voluntary written consent for participation was obtained from either the patient and/or his/her legally authorized representative. All information obtained from the patients was kept confidential and used solely for scientific purposes, and no personal identifiers were disclosed at any point.

Patients were randomly assigned to either the suture or tissue adhesive group using random sampling. Both techniques, including their advantages and limitations, were explained to participants prior to obtaining informed consent. Patients were then allotted for inguinal skin incision closure using either tissue adhesive or sutures, with each technique applied in 35 cases.

After inguinal Lichtenstein tension-free mesh hernioplasty,^3^ skin incision closure was performed using Ethilon 2-0 (RC) in the suture group, while N-butyl-2-cyanoacrylate glue was used in the sutureless group. Patients were shifted to the ward in a hemodynamic stable condition.

### 2.1. Study tools

The first section of the proforma included the patient’s demographic data, type of inguinal surgery, and the method used for wound closure. The second section of the questionnaire recorded details of wound infection, wound dehiscence, and scar appearance. The final section assessed patient and surgeon satisfaction.

The operational definitions of the observations are provided below:

- **Wound infection:** was considered to be present if the patient exhibited redness, swelling, purulent discharge, pain, increased skin temperature, fever, or other systemic signs of infection. These symptoms were assessed until the time of discharge.
- **Complications:** Immediate postoperative complications assessed included hematoma, seroma, allergic dermatitis, and others. Complications were assessed until the patient was discharged.
- **VAS Pain Score:** Postoperative pain experienced by the patient was assessed using the visual analogue scale (VAS) pain score on a scale from 0 to 10 on days 1, 2, 3, 7, 30, and 90.

**Figure:**
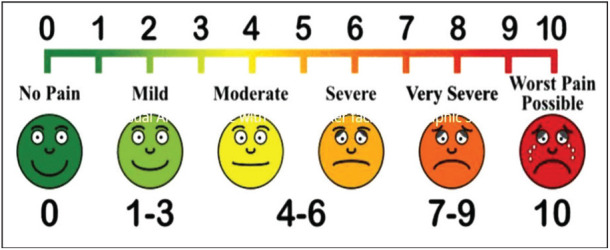
- **Hollander Wound Evaluation Score (HWES):**^6^ Wound dehiscence was measured using the HWES.^6^ The maximum and minimum scores are 6 and 0, respectively. A score of 6 indicates poor wound healing, while 0 indicates very good healing. Wound evaluation was performed on days 1, 2, 3, 7, 30, and 90.
  **Hollander Wound Evaluation Score**^6^

**Figure:**
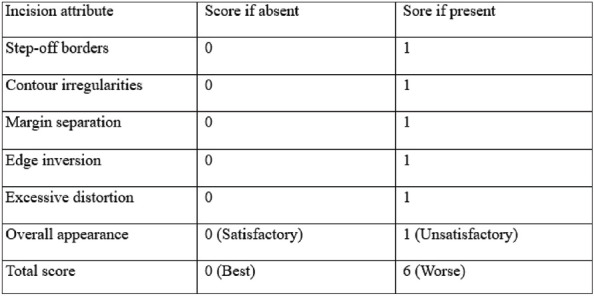
- **Visual Analogue Scar Scale:** Patient perception of the scar was assessed using the visual analogue scar scale. The scale ranges from 0 to 100, where 0 indicates the worst scar and 100 indicates the best possible scar. Evaluations were performed on days 1, 2, 3, 7, 30, and 90.

**Figure:**
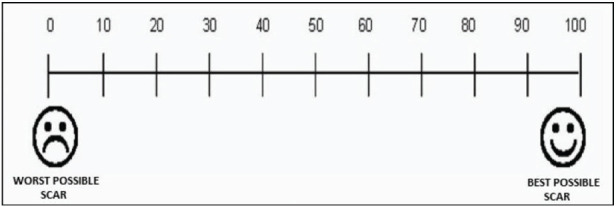
- **Vancouver Scar Scale:**^7^ The scar formed was evaluated using the Vancouver Scar Scale. This scale ranges from 0 to 13, where a score of 0 indicates a good scar and 13 indicates the worst. Assessments were performed on days 1, 2, 3, 7, 30, and 90.

**Figure:**
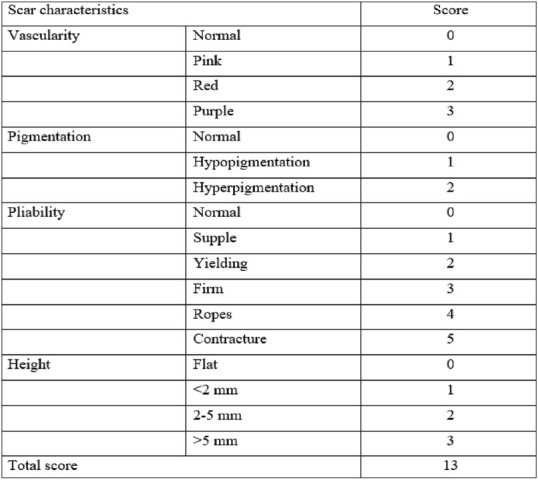
- **Patient Satisfaction Score:** Patient satisfaction was assessed using a standardized 10-point Likert scale, validated in surgical outcome studies for subjective comfort, cosmetic perception, and overall experience. Postoperative satisfaction was evaluated on days 7, 30, and 90.

**Figure:**
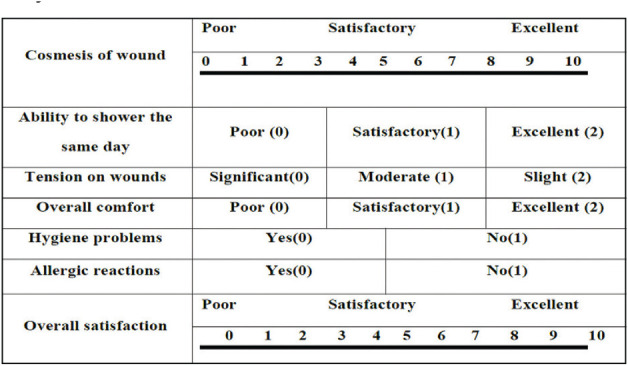
- **Surgeon Satisfaction Score:** Surgeon satisfaction was assessed using a structured 10-point Likert scale reflecting ease of application, reliability of closure, and professional preference. Postoperative satisfaction of the surgeon was evaluated on days 7, 30, and 90.

**Figure:**
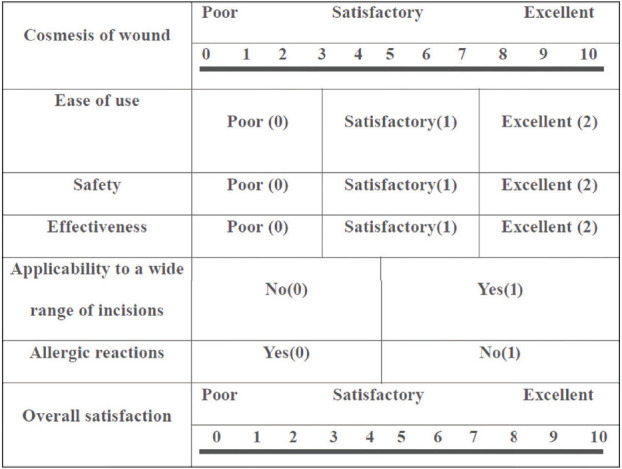

### 2.2. Statistical analysis

All data were entered into Microsoft Excel and subsequently analyzed using SPSS version 25 software. The primary variables, such as age, BMI, wound closure time, and hospital stay, were expressed as means and compared between groups using the unpaired t-test. Secondary outcomes, including postoperative pain (VAS), wound healing (Hollander Score), and scar quality (Vancouver Scar Scale), were expressed as mean ± standard deviation and analyzed using unpaired t-tests. Qualitative variables, such as complication rates and gender distribution, were compared using the chi-square test. A p-value of less than 0.05 (*p* < 0.05) was considered statistically significant.

## 3. RESULTS

A total of 70 patients, i.e., 35 in each group, were recruited for this study. The age distribution of the study participants is presented in Table 1. Most patients belonged to the 18–30 years age group, followed by those aged over 50 years. The age distribution across the two groups was similar and comparable (*p* = 0.835).

There was a higher proportion of males in both groups, with 28 (80.0%) in the suture group and 27 (77.1%) in the *N*-butyl-2-cyanoacrylate group, compared to females. The sex distribution between the two groups was similar and comparable (*p* = 0.770). The majority of the patients had a normal BMI (Body Mass Index), accounting for 85.7% and 80% in Groups 1 and 2, respectively. Less than 15% of cases were overweight or obese. The BMI distribution across the two groups was similar and therefore comparable (*p* = 0.773). All patients had an ASA (American Society of Anesthesiologists) score of either I or II. The distribution of ASA scores between the two groups was comparable, with no statistically significant difference (*p* = 0.596). The most common comorbidities observed were diabetes and hypertension, with similar proportions across both groups. Smoking status was not recorded, which represents a limitation of the study. The proportion of these comorbidities was similar between the groups (*p* = 0.667). Left-sided hernia was more common in Group 1 (42.8%), while right-sided hernia was more common in Group 2 (42.8%); however, this difference was not statistically significant (*p* = 0.459). Indirect hernia was more common than direct hernia in both groups, accounting for 21 cases (60.0%) in the suture group and 19 cases (54.2%) in the N-butyl-2-cyanoacrylate group. The distribution of hernia types was similar between the two groups (*p* = 0.629).

Postoperative pain experienced by patients was evaluated using the VAS score on a Likert scale ranging from 0 to 10. A score closer to 10 indicates severe pain, whereas a score closer to 0 indicates less pain. The pain score was significantly higher in the suture group compared to Group 2 (*N*-butyl-2-cyanoacrylate) during the initial 12 h post-surgery (*p* < 0.05). Therefore, the difference in pain scores between the two groups was not statistically significant at 24 h (*p* = 0.105) or 48 h (*p* = 0.173). The mean time taken (in minutes) for wound closure was 11.7 ± 2.6 min in Group 1 and 8.8 ± 1.4 min in Group 2. The mean taken in Group 2 was lower than in Group 1, and this difference was statistically significant (*p* = 0.000). The presence of wound infection was assessed at 3, 7, 30, and 90 days post-surgery. There was no infection in either group at any time point. One patient in Group 1 presented with a hematoma at the incision site post-surgery. No other complications, such as seroma or allergic dermatitis, were noted among the remaining patients. The mean duration of hospital stay was 3.5 ± 1.6 days in Group 1 and 1.9 ± 0.8 days in Group 2. The difference was statistically significant (*p* = 0.000). The presence of chronic pain at the incision site 30 days post-surgery was observed in six patients in Group 1 and one patient in Group 2. This difference was statistically significant at 30 days (*p* = 0.046). However, on day 90, no pain was reported in either group.

The HWES was assessed for all patients on postoperative days 3, 7, 30, and 90. The score ranges from 0 to 6, with higher scores reflecting poorer wound healing and lower scores indicating better healing. On day 3, the scores of both groups were similar, with no statistically significant difference (*p* = 0.124). On days7 and 30, the scores in Group 2 were lower than those in Group 1, and this difference was statistically significant (*p* < 0.05). However, on day 90, the wound scores were again similar between the two groups (*p* = 0.146) (Table 2). Scar’s outcomes were better in the N-butyl-2-cyanoacrylate group than in the suture group. There was a significant difference in the scar evaluation scores between the two groups based on the Vancouver scale at intervals of 3, 7, and 30 days, but the scores were similar on day 90 (Table 3). The mean patient satisfaction scores of Group 1 and Group 2 were also compared. Scores ranged from 0 to 10, with values closer to 10 indicating higher satisfaction. It was found that Group 2 patients, who underwent wound closure with glue, were more satisfied than those in Group 1. This difference in patient satisfaction was greater in Group 2 at all follow-up intervals, i.e., 7th, 30th, and 90th days (Table 4). Surgeon satisfaction scores for using suture versus glue for wound closure were evaluated on days 7, 30, and 90. Surgeon reported higher satisfaction with the suture method compared to glue at all time intervals. This difference was statistically significant at all intervals (*p* < 0.05) (Table 5).

## 4. DISCUSSION

The findings from this comparative analysis highlight several important considerations in the choice between N-butylcyanoacrylate glue and conventional sutures for skin closure following inguinal hernioplasty.

Postoperative pain assessed using the VAS score showed that the mean pain was greater in Group 1 (suture) compared to Group 2 (N-butyl-2-cyanoacrylate) at 2, 6, and 12 h post-surgery (*p* < 0.05). However, the pain experience at 24 h (*p* = 0.105) and 48 h (*p* = 0.173) was similar between the groups, which is comparable with the findings reported in other studies by Singh et al.^4^ and Dua et al.^5^

No cases of infection were reported in either group during the entire follow-up period. However, Sahu et al.^8^ and Singh et al.^4^ reported a higher incidence of wound infection in the suture group compared to the glue group, although the difference was not statistically significant. Toriumi et al.^9^ reported no infections in both groups, similar to our findings. There was one case of hematoma in the suture group (2.8%), while none in the glue group; however, this difference was not significant. Similar results were reported by Huguenin-Dezot et al.^10^ and Toriumi et al.^9^ No cases of seroma were observed in both the groups in our study. Huguenin-Dezot et al.^10^ reported seroma rates of 0.8% in the suture group and 0% in the glue group.

We observed chronic pain in 17.1% and 2.8% of patients in the suture and glue groups, respectively, at 1-month post-surgery, which was statistically significant. However, at the end of 3 months, the incidence was similar between the groups. Huguenin-Dezot et al.^10^ reported that the incidence of chronic pain at the end of 1 year was similar between the suture group (11%) and the glue group (13%), with no statistically significant difference. Testini et al.^11^ and Jani^12^ compared chronic pain among patients receiving glue versus sutures. The former study found a significant association between mesh fixation and chronic groin pain, whereas the latter study found no significant difference. Therefore, further investigation with long-term follow-up is needed to draw valid conclusions regarding chronic pain.

The mean duration of hospital stay was 3.5 days in the suture group, compared to 1.9 days in the glue group, which was statistically significant. Singh et al.^4^ and Chibbaro and Tacconi^13^ also reported similar findings. This difference can be attributed to faster wound closure, reduced postoperative pain, and earlier mobilization in the tissue adhesive group, which facilitated quicker discharge.

We compared wound healing using the HWES, which was significantly lower in the glue group than in the suture group at both the 7th day and the 30th day post-surgery. Singh et al.^4^ also reported similar results. Ong et al.^14^, however, found that suboptimal scores (<5) were more frequent in the glue group than in the suture group, although the difference was not statistically significant. Bernard et al.^15^ similarly reported no significant difference in HWES at 2 months.

Scar outcomes were better in the cyanoacrylate group than in the suture group, with a significant difference in the scar evaluation score based on the Vancouver scale at 3, 7, and 30 days post-surgery; however, the scores were similar on day 90. Dua et al.^5^ also reported poorer scar scores in the suture group than the tissue glue group based on the Vancouver Scar Scale. However, Daykan et al.^16^ found similar Observer Scar Assessment scores between the glue and suture groups. Similarly, Singer et al.^17^ reported cosmetic appearance to be similar at 3 months. Cosmetic outcomes were consistently superior in the glue group, with smoother scars and less tissue distortion.

In our study, patient perception of wound scars, assessed using the VAS, showed that patients receiving glue (*N*-butyl-2-cyanoacrylate) for wound closure were more satisfied with the appearance of their incision site scars than those in the conventional suture group at 3, 7, and 30 days post-surgery, with significantly higher VAS scores (*p* < 0.05). On day 90, patients in the glue group reported higher scores than those in Group 1, but the difference was not statistically significant. Similarly, Toriumi et al.^9^ observed comparable results. However, Ong et al.^14^ compared the two methods for pediatric surgical incisions and found the VAS scar scores to be similar between the suture and glue groups (*p* = 0.68), as reported by the children’s parents. Even Daykan et al.^16^ found that patient scar assessments were similar between the glue and suture groups. In contrast, Bernard et al.^15^ reported that the mean VAS scar score was higher in the suture group (63.3) than in the glue group (47.8), and this difference was statistically significant. This was the only study in which patients reported sutures to be better than glue. However, the study was conducted among children and adolescents, and the scar perceptions were reported by their parents, which could possibly explain this finding.

The mean time taken for wound closure was shorter with tissue glue compared to sutures, and this difference was statistically significant. Singh et al.^4^, Dua et al.^5^, and Sahu et al.^8^ also reported that the mean closure time was significantly shorter for the glue group than for the suture group. In contrast, Ong et al.^14^ observed no difference in incision closure time between the two methods in the pediatric age group. Differences in patient populations and the type of surgical incision across studies make comparisons and interpretations challenging.

In our study, patient satisfaction scores were higher among patients whose wounds were closed using the glue technique compared to sutures at all follow-up intervals. Overall, most studies by Chibbaro and Tacconi,^13^ Toriumi et al.^9^, and Shivamurthy et al.^18^ also reported higher patient satisfaction with glue than with suture.

In our study, surgeon satisfaction scores were higher with the suture method compared to glue at all follow-up intervals, and this difference was statistically significant. Shivamurthy et al.^18^ however, found no significant difference in mean surgeon satisfaction between glue and suture. Most other studies have not compared the surgeon satisfaction for skin closure, likely because it depends on the individual surgeon’s skill and experience with either glue or sutures.

*N*-Butyl-2-cyanoacrylate glue is generally well tolerated. Reported side effects in the literature include mild local dermatitis, allergic reactions, and rare cases of wound dehiscence in high-tension closures. In our study, no significant adverse effects were observed, supporting its safety profile for inguinal skin incision closure. However, its tensile strength is lower, making it less suitable for high-tension closures or for patients with compromised wound healing. Additionally, the cost of tissue adhesives remains higher.

The strengths of this study include its prospective design and the use of multiple validated clinical assessment tools, including the VAS for pain, the HWES for healing, and the Vancouver Scar Scale for aesthetic outcomes. Additionally, random sampling was employed to minimize selection bias.

Limitations of the study: This study has several limitations, including its single-center design and a modest sample size, which may limit generalizability. Follow-up was limited to 3 months, and surgeon satisfaction scores may be inherently subjective. Future multicenter studies with larger sample sizes and long-term follow-up periods could further validate these findings.

## 5. CONCLUSION

Tissue glue (*N*-butyl-2-cyanoacrylate) has been found to be superior to sutures for wound closure in inguinal hernia surgery in terms of reduced postoperative pain, shorter wound closure time, improved scar appearance, decreased hospital stays, and a lower risk of chronic pain at the incision site. It is a safe and effective alternative to sutures, particularly when patient comfort and cosmetic outcomes are prioritized.

## AUTHORS’ CONTRIBUTION

NDS: Conceptualization, Methodology, Writing—original draft preparation, Writing—review and editing. AK: Conceptualization, Methodology, Formal analysis, Data curation, Writing—original draft preparation, Writing—review and editing, Supervision. ASD: Writing—review and editing, Supervision. All authors contributed to the study conception and design. All authors read and approved the final manuscript.

## ETHICAL CONSIDERATIONS

The study was conducted only after obtaining approval from the Ethics Committee (No: IEC/2023/178). Before including patients in the study, written informed consent was obtained from the patient and/or his/her legally authorized representative. Information obtained from the patients was kept confidential and used solely for scientific purposes. No personal identity was disclosed at any point in time.

## CONFLICT OF INTEREST

The authors have no conflicts of interest to declare.

## DATA AVAILABILITY STATEMENT

Data will be available upon reasonable request from the corresponding author.

## Figures and Tables

**Table 1.** Age distribution of study groups (*N* = 70).

Age group (years)	Group 1 (suture)	Group 2 (*N*-butyl-2-cyanoacrylate)	Chi-square (*p*-value)
18–30	13 (37.1%)	10 (28.5%)	
31–40	7 (20.0%)	8 (22.8%)	0.858 (0.835)
41–50	4 (11.4%)	6 (17.1%)	
>50	11 (31.4%)	11 (31.5%)	
Total	35 (100%)	35 (100%)	

**Table 2.** Mean Hollander Wound Evaluation Score (HWES) (out of 6) at various time intervals among the study groups (*N* = 70).

Time interval (in days)	Group 1 (*N* = 35) (suture)	Group 2 (*N* = 35) (*N*-butyl-2-cyanoacrylate)	t-test (*p*-value)
3	4.7 ± 0.3	4.5 ± 0.7	1.55 (0.124)
7	3.5 ± 1.2	2.6 ± 0.4	4.20 (0.000*)
30	1.2 ± 0.8	0.9 ± 0.3	2.07 (0.041*)
90	0.7 ± 0.4	0.9 ± 0.7	1.46 (0.146)

*Statistically significant.

**Table 3.** Scar evaluation at various intervals using the Vancouver scar scale (0–13) among the study groups (*N* = 70).

Time interval (in days)	Group 1 (*N* = 35) (suture)	Group 2 (*N* = 35) (*N*-butyl-2-cyanoacrylate)	*t*-test (*p*-value)
3	4.4 ± 2.1	3.4 ± 1.3	2.08 (0.040*)
7	3.2 ±0.9	1.9 ± 0.8	6.38 (0.000*)
30	0.8 ± 1.0	0.7 ± 0.6	5.58 (0.000*)
90	0.7 ± 0.5	0.7 ± 0.1	0.00 (1.002)

*Statistically significant.

**Table 4.** Mean patient satisfaction score (0–10) at various intervals among the study groups (*N* = 70).

Time interval (in days)	Group 1 (*N* = 35) (suture)	Group 2 (*N* = 35) (*N*-butyl-2-cyanoacrylate)	t-test (*p*-value)
7	5.6 ± 2.4	7.4 ±1.2	3.96 (0.000*)
30	8.5 ± 1.2	9.2 ± 0.6	3.08 (0.002*)
90	9.1 ± 0.9	9.8 ± 0.4	4.20 (0.000*)

*Statistically significant.

**Table 5.** Mean surgeon satisfaction score (0–10) at various intervals among the study groups (*N* = 70).

Time interval (in days)	Group 1 (*N* = 35) (suture)	Group 2 (*N* = 35) (*N*-butyl-2-cyanoacrylate)	*p*-value
7	9.5 ± 1.2	7.1 ± 1.4	7.77 (0.000*)
30	9.8 ± 1.1	8.4 ± 1.3	4.86 (0.000*)
90	9.7 ± 1.1	8.9 ± 0.9	3.33 (0.001*)

*Statistically significant.
